# Acute transfusion-related abdominal injury in trauma patients: a case report

**DOI:** 10.1186/s13256-016-1075-4

**Published:** 2016-10-19

**Authors:** P. Michel, D. Wähnert, M. Freistühler, M. G. Laukoetter, S. Rehberg, M. J. Raschke, P. Garcia

**Affiliations:** 1 Department of Trauma-, Hand- and Reconstructive Surgery, Westfaelische Wilhelms University Muenster, Waldeyer Str. 1, 48149 Muenster, Germany; 2 Department of General and Visceral Surgery, Westfaelische Wilhelms University Muenster, Muenster, Germany; 3 Department of Anesthesiology, Anesthesia, Intensive Care, Emergency and Pain Medicine, University Medicine Greifswald, Greifswald, Germany

**Keywords:** Trauma, Abdominal compartment, Transfusion, Resuscitation

## Abstract

**Background:**

Secondary abdominal compartment syndrome is well known as a life-threatening complication in critically ill patients in an intensive care unit. Massive crystalloid fluid resuscitation has been identified as the most important risk factor. The time interval from hospital admittance to the development of manifest abdominal compartment syndrome is usually greater than 24 hours. In the absence of any direct abdominal trauma, we observed a rapidly evolving secondary abdominal compartment syndrome shortly after hospital admittance associated with massive transfusion of blood products and only moderate crystalloid resuscitation.

**Case presentation:**

We report the case of an acute secondary abdominal compartment syndrome developing within 3 to 4 hours in a 74-year-old polytraumatized white woman. Although multiple fractures of her extremities and a B-type pelvic ring fracture were diagnosed by a full body computed tomography scan, no intra-abdominal injury could be detected. Hemorrhagic shock with a drop in her hemoglobin level to 5.7 g/dl was treated by massive transfusion of blood products and high doses of catecholamines. Shortly afterwards, her pulmonary gas exchange progressively deteriorated and mechanical ventilation became almost impossible with peak airway pressures of up to 60 cmH_2_O. Her abdomen appeared rigid and tense accompanied by a progressive hemodynamic decompensation necessitating mechanic cardiopulmonary resuscitation. Although preoperative computed tomography scans showed no signs of intra-abdominal fluid, a decompressive laparotomy under cardiopulmonary resuscitation conditions was performed and 2 liters of ascites-like fluid disgorged. Her hemodynamics and pulmonary ventilation improved immediately.

**Conclusions:**

This case report describes for the first time acute secondary abdominal compartment syndrome in a trauma patient, evolving in a very short time period. We hypothesize that the massive transfusion of blood products along with high doses of catecholamines triggered the acute development of abdominal compartment syndrome. Trauma teams need to consider a rapidly developing secondary abdominal compartment syndrome to be a potential cause of hemodynamic decompensation not only in the later phase of treatment but also in the emergency phase of treatment.

## Background

Abdominal compartment syndrome (ACS) is a well-known complication in a wide variety of critically ill patients. It is characterized by a potentially life-threatening elevation of intra-abdominal pressure (IAP) and subsequent deterioration of organ function [1]. The increased capillary permeability and hydrostatic pressure during the initial phase of posttraumatic shock is the driving force for interstitial fluid accumulation [2]. Increased IAP leads to an impaired perfusion with subsequent ischemia of the intestines and other peritoneal and retroperitoneal organs [3]. The World Society of the Abdominal Compartment Syndrome defines ACS as a sustained IAP greater than 20 mmHg [4]. IAP can be measured indirectly via a urine catheter. The only curative treatment is a decompressive laparotomy and a temporary open abdominal cavity [5, 6].

Depending on the underlying cause, primary ACS can be differentiated from secondary ACS. Primary ACS is defined by an intra-abdominal process, such as acute intra-abdominal bleeding that directly increases the IAP. Secondary ACS in contrast is a complication found in patients without primary abdominal injury [7]. Several risk factors for the development of ACS in critically ill patients have been identified, such as hypothermia, acidosis, body mass index, sepsis, mechanical ventilation, massive fluid resuscitation, multiple transfusions, abdominal trauma, and abdominal surgery [8].

According to the literature, the timespan for the development of ACS is usually 24 hours or more after admittance to a hospital [9]. In trauma patients, the mortality rate of ACS is very high and ranges from 25 to 75 % [3, 10]. Therefore, the reduction of risk factors and early recognition of ACS are crucial to optimize clinical outcome.

## Case presentation

We report the case of a 74-year-old white woman who was hit as a pedestrian by a car moving at approximately 40 to 50 km/hour. The initial emergency physician started treatment according to standard protocols. Her cervical spine was immobilized with a stiff collar and a pelvic compression belt was applied anticipating a pelvic injury. With a Glasgow Coma Scale of 14 at that time, there was no need for an emergency intubation. Her vital signs were documented; she had an arterial blood pressure of 160/100 mmHg and a heart rate of 86 beats per minute, indicating a negative shock index.

During transport she received 2×500 ml of crystalloid fluids (Sterofundin; B. Braun Melsungen, Germany). At our emergency department a multidisciplinary team of trauma surgeons, anesthesiologists, a neurosurgeon, a visceral surgeon, and a radiologist was awaiting the patient. At the time of her arrival, she was still awake and responsive with a Glasgow Coma Scale of 14. A physical examination according to the guidelines of Advanced Trauma Life Support showed no signs for an airway, breathing, or circulation problem (ABC-problem). Her thorax was stable and there were no indications for deterioration in gas-exchange. The pelvic splint applied by the emergency physician was left in place. An abdominal examination by a trained visceral surgeon showed no signs of tension or muscular defense. There were no visible abdominal contusion marks. Numerous fractures of her extremities were suspected, which were immediately repositioned and splinted. A focused assessment with sonography for trauma indicated no pleural effusion or pericardial effusion, and no free abdominal fluid, air, or hints for organ lacerations. A thoracic X-ray showed no signs of a pneumothorax or rib fractures. After a pelvic X-ray, non-dislocated fractures of the left ramus superior and inferior of her os pubis were suspected. The first blood gas analysis in our emergency room revealed a hemoglobin (Hb) level of 10.6 g/dl and a lactate level in physiological range (1.4 mmol/l). After initial assessment of our patient, a full body trauma computed tomography (CT) scan with the application of a radiocontrast agent was performed and the following diagnoses were stated using fracture classifications according to the Association for the Study of Internal Fixation (AO):

- Traumatic subarachnoid hemorrhage
- Tripod fracture on the right side with orbital floor affection
- Three-part fracture of her proximal humerus on the left side (AO 11 C3)
- Two-part fracture of her proximal humerus on the right side (AO 11 C2)
- Small intracapsular hematoma of her spleen
- Pelvic ring fracture (AO type B)
  - ○ Fracture of the left ramus superior and inferior of her os pubis
  - ○ Longitudinal fracture of her right sacrum
- Pertrochanteric femur fracture right (AO 32 A1)
- Fracture of her left proximal tibia (AO 42-C3)

There were no signs for free abdominal fluid or air, no signs for the extravasation of the radiocontrast agent in her thoracic, abdominal, or pelvic cavity (indicating a vascular injury), and no signs for a greater hematoma in the small pelvis. Her medical history revealed several internal comorbidities but no signs of coagulopathy or anticoagulant medication.

**Fig. 1:**
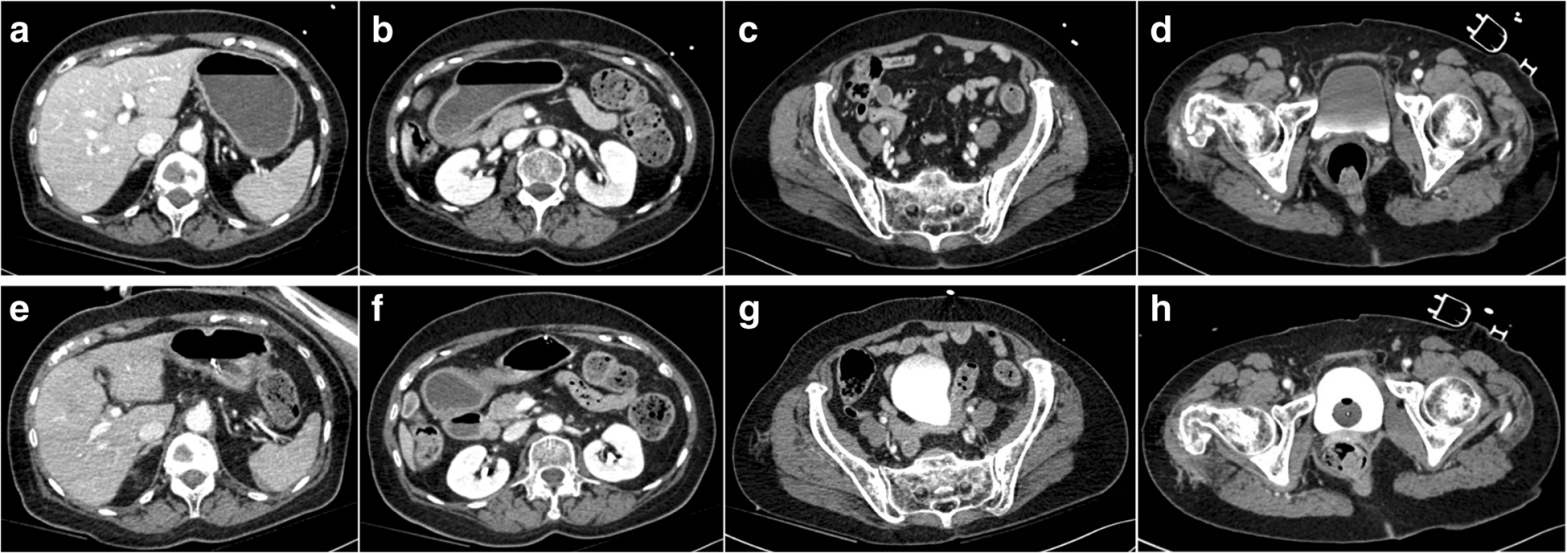
*Upper series* (**a**-**d**): First trauma computed tomography scan without any signs for free abdominal fluid or air, no signs for the extravasation of the radiocontrast agent in the abdominal or pelvic cavity (indicating a vascular injury), and no signs for a greater hematoma in the small pelvis. *Lower series* (**e**-**h**): Second trauma computed tomography scan 90 minutes later still revealed no free abdominal fluid or air, no signs of extravasation of the radiocontrast agent in the thoracic, abdominal, or pelvic cavity, and no signs for a progressing hematoma of the spleen or in the small pelvis

During the diagnostic phase she remained awake and neurologically adequate with stable hemodynamics. According to the concept of damage control the indication for external fixation of her pelvis and her lower extremity was made and she was transferred to our intensive care unit (ICU) before early operative treatment. In the course of the preoperative preparations she presented signs of respiratory exhaustion with increasing hypoxia and had to be intubated. During the intubation her clinical condition worsened dramatically due to aspiration and hemodynamic decompensation. Stabilization was only achieved after a period of approximately 5 minutes of mechanic cardiopulmonary resuscitation (CPR) and continuous infusions of catecholamines. A physical examination of her abdomen showed no signs of tension. An immediate blood gas analysis revealed an Hb level of 5.7 g/dl; her lactate level was still in physiological range (0.5 mmol/l). An additional CT scan of her thorax, abdomen, and pelvis was performed due to the suspected diagnosis of anemic shock in order to rule out new sources of bleeding after the CPR. However, the second CT scan showed no new findings; explicitly, no free abdominal fluid or air, no signs of extravasation of the radiocontrast agent in her thoracic, abdominal, or pelvic cavity, and no signs for a progressing hematoma of her spleen or in her small pelvis (Fig. 1).

**Fig. 2:**
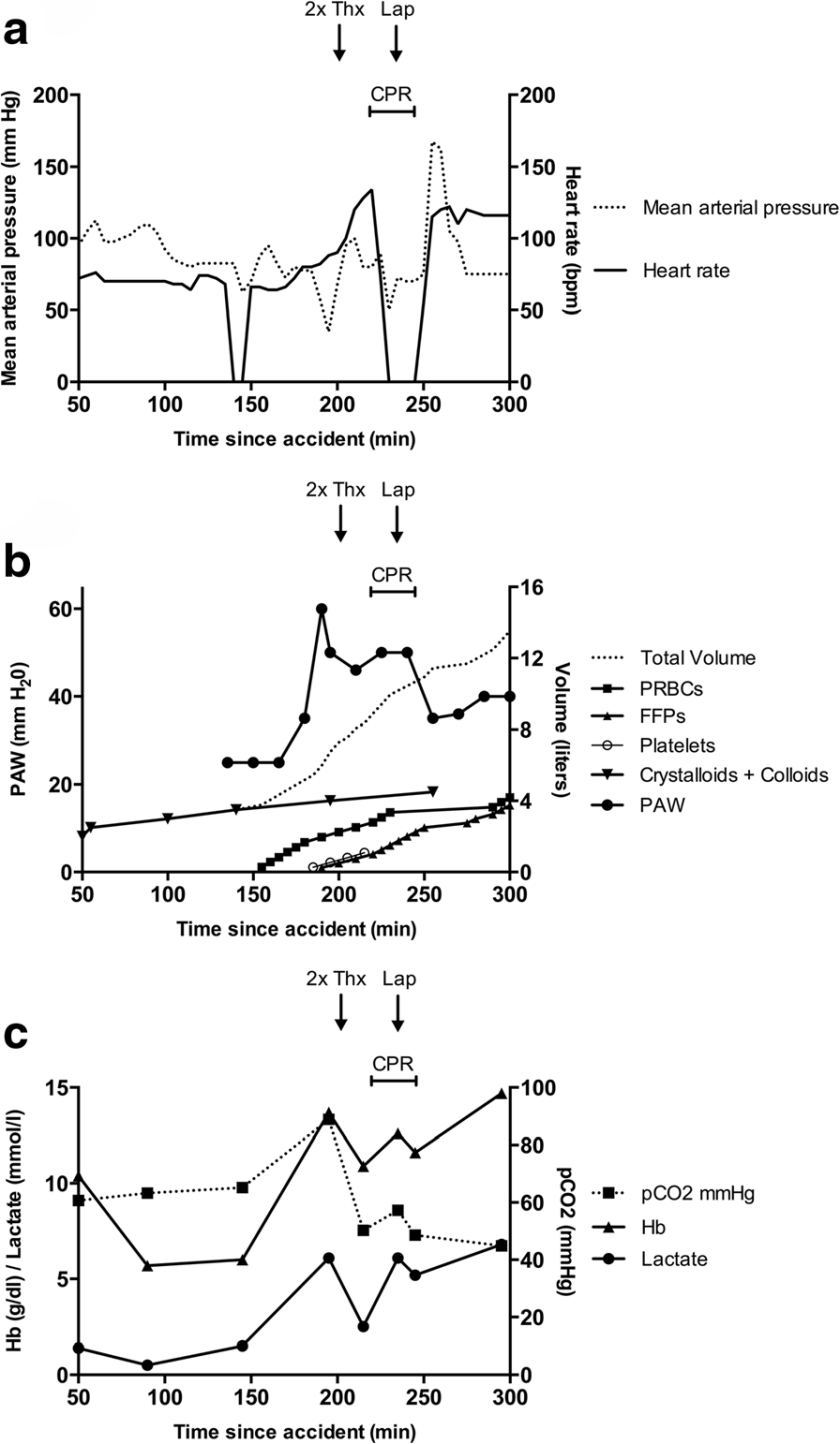
Key parameters of shock before and after decompressive laparotomy. **a** Vital parameters (medium blood pressure and pulse) prior to the cardiopulmonary resuscitation and decompressive laparotomy. **b** The peak airway pressure took a critical rise shortly after starting the massive transfusion of packed red blood cells and fresh frozen plasma. **c** The course of the hemoglobin level and partial pressure of carbon dioxide are reversed shortly after the decompressive laparotomy. *Bpm* beats per minute, *CPR* cardiopulmonary resuscitation, *FFPs* fresh frozen plasma, *Hb* hemoglobin, *Lap* laparotomy, *min* minute, *PAW* peak airway pressure, *pCO*_*2*_ partial pressure of carbon dioxide, *PRBCs* packed red blood cells, *Thx* chest drain

She had received 5×500 ml of crystalloid fluid (2.5 liters) and 2×500 ml of colloidal fluid (1 liter) so far. According to clinical transfusion protocols, from this point of time on she received packed red blood cells (PRBCs), platelets, and fresh frozen plasma (FFPs) to compensate for the sudden drop in her Hb level. Within 25 minutes the first six PRBCs were transfused. In addition, she required high doses of catecholamines (Fig. 2a).

After the second CT scan, she was brought immediately to our operating room to stabilize her pelvic ring fracture. At the start of the operation, her ventilation situation worsened dramatically, with hypercapnia, hypoxia, and an increasing peak airway pressure of up to 60 cmH_2_O (Fig. 2b). In parallel, she presented an upper venous congestion with subsequent hemodynamic decompensation, necessitating mechanic CPR. Her abdomen appeared increasingly rigid and tense. Her lactate level was now increasing to 6.8 mmol/l (Fig. 2c). Bilateral chest drainages were applied to rule out a hemopneumothorax, but pulmonary ventilation became almost impossible. At the same time, a cardial tamponade was excluded by transesophageal echocardiography, and bronchoscopic evaluation of her airways showed no pathological findings. Under ongoing CPR, a pelvic clamp was applied to her dorsal pelvic ring for mechanical stabilization before performing a decompressive laparotomy. After opening her peritoneum, 2 liters of clear ascites-like fluid could be removed. After opening her abdomen widely, her ventilation situation immediately improved and her peak airway pressure was lowered to 35 cmH_2_O. After a total of 12 minutes of CPR a sinus rhythm and hemodynamic stabilization could be retained again. Catecholamine doses could be reduced extensively. She received 12 PRBCs, eight FFPs, and four platelets prior to the decompressive laparotomy. Combined with crystalloids and colloids, this equals a total volume of 10.5 liters of resuscitation fluids.

A detailed inspection of her abdominal cavity by a specialized visceral surgeon revealed no major bleeding or organ injury. Her spleen showed a small intracapsular hematoma, but no signs of greater injury. Her liver appeared to be congested. Her bowel looked edematous and showed signs of the beginning of ischemia. An inspection of her small pelvis showed no signs of bleeding. As a precaution, her pelvis was packed and the laparotomy was temporarily closed with a low-adherent wound contact layer, consisting of a flexible polyamide net coated with soft silicone.

In the postoperative course of treatment, additional operations followed. Her abdomen was closed 23 days after the decompressive laparotomy. She was transferred from our ICU to our intermediate care unit 40 days after trauma. She was transferred to a neurological rehabilitation center 8 weeks after trauma.

## Discussion

Secondary ACS has been described in trauma and burn patients receiving high amounts of crystalloid resuscitation. Most studies describe the development of secondary ACS in trauma patients with high volumes of crystalloids after at least 24 hours in an ICU. Kasotakis *et al*. [9] showed that the volume of crystalloid resuscitation within the first 24 hours was associated with the development of ACS in a dose-dependent fashion. Patients who received 10 to 15 liters of crystalloids in the first 24 hours after injury had 5 times the odds to develop an ACS compared to the group who received only 5 to 10 liters. There even was a ninefold risk for patients receiving more than 15 liters of crystalloids [9].

In contrast, we observed the development of a manifest secondary ACS with only 3.5 liters of crystalloid/colloid resuscitation prior to a decompressive laparotomy approximately 3 hours after admittance to our hospital. We hypothesize that the massive transfusion of blood products combined with the high doses of catecholamines were responsible for the early onset of ACS.

The unifying feature of secondary ACS appears to be the presence of shock requiring aggressive resuscitation. This has been described by numerous authors before and has led, among other reasons, to a more restrictive use of crystalloid fluids in emergency medicine [11]. As a possible pathophysiological explanation, we consider a combination of the following factors to be responsible. Our patient experienced a severe hemorrhagic shock with a drop in Hb level to 5.7 g/dl, which led to a systemic vasoconstriction to maintain organ perfusion. This response is known to be disproportionately larger within the splanchnic circulatory system. Approximately 40 % of the total systemic vascular resistance increase during cardiogenic shock is due to mesenteric vasoconstriction [12]. This effect is further aggravated by a high-dose application of catecholamines through adrenergic receptor-mediated vasoconstriction [13]. The prolonged hypoperfusion results in mesenteric ischemia. After restoring circulation, the reperfusion injury leads to increased microvascular permeability with vascular leakage and bowel edema [14]. The gut is especially susceptible to global ischemia and shock-induced low-flow states. Bowel ischemia with consequent reperfusion leads to increased microvascular permeability resulting in bowel edema [15]. This was also documented by the visceral surgeon, who performed the detailed inspection of our patient’s abdominal cavity. The reperfusion injury was further aggravated by the massive transfusion of blood products. Our patient received 12 PRBCs, eight FFPs, and four platelets with the working diagnosis of hemorrhagic shock. Combined with crystalloids and colloids, this equals a total volume of 10.5 liters of resuscitation fluids prior to the decompressive laparotomy. This was chronologically directly associated with an increasing abdominal tension, an increasing peak airway pressure, and rising lactate levels. Notably, we did not see a rise in her lactate level with the start of the catecholamine application, but we did see a rise in her lactate level shortly after a suspected increase in her IAP. Immediately after decompressive laparotomy, her peak airway pressure and lactate levels decreased, hemodynamic stabilization could be achieved, and catecholamine doses could be reduced. It is remarkable that both CT scans showed no signs of free abdominal fluid, whereas 2 liters of ascites-like fluid were disgorged only 60 minutes later.

According to the outlined pathophysiological concept, the largest amount of resuscitation volume had to be applied just when the intestine was most vulnerable to the reperfusion injury, which led to a rapidly developing secondary ACS.

A recent study supports our hypothesis that the transfusion of blood products triggered the secondary ACS. Hwabejire et al. showed that the risk of ACS and associated mortality starts to rise after administration of 96 ml resuscitation fluid per kg bodyweight during resuscitation for blunt traumatic hemorrhagic shock. Special attention should be paid to the fact that no difference between crystalloids and blood products could be detected. Irrespective of the type of resuscitative fluid administered, the total volume of fluids correlates with ACS risk. In our not-representative case, we saw the start of symptoms for a rising IAP after passing the inflection point of 6 to 7 liters of total administered volume proposed by Hwabejire *et al*. [16].

A possible differential diagnosis is fat embolism, which can be a reason for sudden deterioration of the respiratory system. The immediate improvement of our patient’s symptoms after the decompressive laparotomy favors secondary ACS.

Retrospectively, the most likely explanation for her extensive hemorrhagic shock was a combination of the numerous extremity fractures together with her pelvic ring fracture. She had humeral head fractures on both sides, a pertrochanteric fracture of her right femur, and a fracture of her left proximal tibia. These four fractured long bones alone can account for a significant blood loss. Both CT scans of her thorax, abdomen, and pelvis showed no signs of an organ injury or any major bleeding source. We did not detect any signs of extravasation of the radiocontrast agent. The minor hematoma accompanying her pelvic ring fracture did not progress between the first and second CT scans. Therefore, we did not consider an angiography or even pelvic packing in our emergency room to be necessary and left the pelvic splint applied by the emergency physician in place. The minor delay until the start of operative treatment was due to capacity reasons, as all operating rooms of our department were running. An emergency stabilization of her pelvis most probably would not have prevented her deterioration at that point in time.

A limitation of this case report is the missing actual IAP provided by a urine catheter measurement. Because this was an emergency situation, there was no time for this routine measurement. The above-mentioned clinical symptoms for ACS were obvious and rapidly developing. Although it is standard in an ICU, the measurement of her IAP would possibly have delayed the decompressive laparotomy in this trauma setting.

From the current literature the pathophysiological influence of crystalloid/blood transfusion and catecholamine usage cannot be differentiated. Further studies are needed to analyze whether this is just a volume-based effect, or whether the combined use of blood products and high-dose catecholamines specifically triggers the rapid development of secondary ACS.

The following factors should raise the trauma teams’ awareness for secondary ACS in the absence of any abdominal injuries [5, 6, 16]:

- Rapidly rising peak airway pressure and progressive hemodynamic destabilization
- Tense abdomen in the clinical examination
- Resuscitation volume of ≥100 ml per kg body
- Sustained IAP >20 mmHg in urine bladder measurement

## Conclusions

In summary, we report a case of rapidly developing secondary ACS in a polytraumatized patient only 60 minutes after the transfusion of blood products started and 3.5 hours after initial trauma. Acute secondary ACS is a critical complication in trauma patients; it is associated with high resuscitation volumes of crystalloids and blood products in combination with high-dose usage of catecholamines. Progressive deterioration of the ventilation pressure without any signs of acute bleeding after massive transfusion can be symptoms of ACS. ACS is a life-threatening condition and the only effective therapy is early decompressive laparotomy. Anticipation of ACS is not only important at later stages with the patient in an ICU, but also in the very early phase after admission to hospital. However, prevention of an ACS must be the primary goal of therapy. Restrictive transfusion protocols may help to reduce the incidence of ACS and might be further adjusted in the future.

## Abbreviations

ACS: Abdominal compartment syndrome
AO: Association for the Study of Internal Fixation
CPR: Cardiopulmonary resuscitation
CT: Computed tomography
FFP: Fresh frozen plasma
Hb: Hemoglobin
IAP: Intra-abdominal pressure
ICU: Intensive care unit
PRBC: Packed red blood cells
